# An Optimal Decision Support System Based on Crop Dynamic Model for N-Fertilizer Treatment

**DOI:** 10.3390/s22197613

**Published:** 2022-10-08

**Authors:** Abhaya Pal Singh, Amol Yerudkar, Davide Liuzza, Yang Liu, Luigi Glielmo

**Affiliations:** 1 Department of Engineering, University of Sannio, 82100 Benevento, Italy; 2 ENEA Fusion and Nuclear Safety Department, 00044 Frascati, Italy; 3 College of Mathematics and Computer Science, and College of Mathematical Medicine, Zhejiang Normal University, Jinhua 321004, China; 4 Jinhua Intelligent Manufacturing Research Institute, Jinhua 321032, China

**Keywords:** precision agriculture, rice, optimization, N-fertilizer, sensors, crop growth dynamics

## Abstract

The efficient handling of nitrogen has become a critical issue in modern agriculture, from a financial standpoint, as well as in regard to reducing the environmental impacts of using an excessive amount of nitrogen fertilizer. Manure compost is useful for maintaining or raising soil chemical levels without excessive ${\mathrm{NO}}_{3}^{-}$ accumulation; however, for the best grain yield, it should be combined with N fertilizer. Via this study, we aimed to develop an optimal decision support system that indicates when to initiate fertilization based on nitrogen-limited (N-limited) crop growth dynamics. An optimal nitrogen fertilizer (N-fertilizer) management system increases crop yield while maintaining a balance between fertilizer supply and crop demand. This study used the N-limited crop growth model (LINTUL3) to develop an optimal decision support system. In this work, we formulated and resolved two optimization challenges: (i) maximization of biomass growth; and (ii) maximization of growth with the least cost paid on N-fertilizer and its application. Furthermore, two case studies were developed based on the number of fields: (i) optimization for a single field, and (ii) optimization for multiple fields. In the case of multiple fields, it is hypothesized that a fertilizer treatment for one field can leak to other fields and affect the nitrogen dynamics of different fields. Finally, numerical simulations were carried out supporting the theory developed in the paper. The simulations showed that when the proposed work was employed to achieve the goal of optimal nitrogen management for a crop, a 28% to 53% increase in biomass growth under certain scenarios was attained.

## 1. Introduction

The precise management of nitrogen has emerged as the most critical issue in modern agriculture, not only from a cost perspective but also in regard to mitigating the environmental impacts of utilizing excessive nitrogen fertilizer. According to the precision agriculture (PA) hypothesis, both farmers and the environment benefit from altering the management and application of nitrogen fertilizer within individual fields [1]. The economic and environmental advantages of site-specific nitrogen applications can therefore be assessed on the basis of site-specific yield response functions. The overall objective in PA is to improve the quality of food production while simultaneously minimizing costs and mitigating the environmental impact [2] (for example, by reducing the amount of water and fertilizer used). Plants need nitrogen in large quantities [3], and the grower must manage such uptake in order to maintain crop growth. The timing of the nitrogen fertilizer application (to obtain the maximum output) is extremely important [4]. The aim of this article was to develop an optimal decision support system that can inform when and how much nitrogen fertilizer should be applied.

The management of nitrogen in flood-irrigated rice crops is the target of this paper. In flood-irrigated rice, the growers typically forecast the nitrogen fertilizer for the season [5], starting from day 1 to the final growth stage.

Nitrogen fertilizer is more likely to be lost by denitrification in flood-irrigated rice [6], where the fraction of applied nitrogen that is really absorbed by the plants is about 40%, such that 60% of the nitrogen applied to the crops is lost [7] and impacts the greenhouse gases and the groundwater.

There are various studies present in the literature on nitrogen management for the rice field. Article [8] suggested a wet–dry cycle and regulated irrigation increased rice yield. According to their investigation, rice yield and nitrogen fertilizer-use efficiencies were high when nitrogen was applied at 200–250 kg/ha. This article also suggests further optimizing/improving paddy field water; fertilizer management is also required. Biochar and inorganic fertilizer, when used together, have been shown to boost soil fertility and increase crop output [9]. In the paper, various application strategies (270–360 kgN/ha) for the fertilizers have been presented, and according to this, using 60t/ha of biochar together with 270kgN/ha of nitrogen is a potential choice to enhance soil quality and boost the photosynthetic, yield, and yield-related characteristics of noodle rice. A study was carried out in [10], suggesting that the maximum growth in the rice crop can be attained by using 225 kgN/ha. Biochar adoption methodologies for soil quality improvement can be found in [11,12].

Split nitrogen fertilization has been proven to be effective in the rice plantation system. A split application of nitrogen fertilizer is presented in [13], which shows that the grain yield rises as the split application is increased to the application of 300kgN/ha N-fertilizer at various stages. Another approach to the split application of the fertilizer is presented in [14], which describes five field trials with irrigated rice conducted at the International Rice Research Institute (IRRI) between 1991 and 1993, with nitrogen levels ranging from 0 to 400 kg/ha, with splits and timings of application varying. Another method described in [15] investigates the coupling of rice with fish for yields and soil fertility and finds that through complimentary and synergistic interactions between fish and rice plants, rice–fish systems can maximize the benefits of limited land and water resources.

Optimization of nitrogen fertilizer is a necessity in rice crop growth. In the article [16], the authors used a dynamic model of crop growth, i.e., ORYZA-0, to numerically optimize the nitrogen application to rice, giving a maximum grain yield. A nitrogen supply optimization technique was discussed in [17] to boost rice production by managing yield formation factors. An interesting optimization strategy was used in [18], to determine the nitrogen fertilization level optimization approach based on an analysis of the trade-offs between rice production and greenhouse gas emissions.

It is important to note that N production is an energy-intensive industrial activity, whose costs are strongly influenced by the energy market [19,20].

Over the last five years, the percentage of cost versus income has risen, implying that the current system is no longer an option for ensuring the sustainability and competitiveness of rice production. The problem is addressed in [21] for the Malaysian system, but analogous issues hold globally. For instance, the cost of production per hectare for rice agriculture increased by 24% between 2015 and 2017 at FELCRA Seberang Perak [22]. Therefore, there is an urgent need for technological advancements, such as a decision support system (DSS), to modernize rice farming in order to ensure the availability of nitrogen fertilizer and the efficacy of rice production. DSS enables farmers to regulate the variability in plant development across fields, estimate nutrient needs, and apply site-specific inputs. For more details on the economic analysis, please see [23,24].

It is evident from the existing literature that splitting nitrogen fertilization encourages reduced denitrification and that managing nitrogen effectively encourages plant development. In order to establish an optimal decision support system for the application of N-fertilizer in the crop field, the contribution of this work was to connect the notion of splitting the N-fertilizer application with optimization, allowing for the development of a more effective system. This work can be used in a fully automated manner or manually to decide on how much fertilizer is to be applied. In the event that the system is entirely automated, sensors should be installed to monitor the nitrogen stress and, based on that, carry out the treatments.

The following notations are used throughout the paper:

- A binary variable b∈B:={1,0}, which indicates to use fertilizer when b=1 and to not do anything when b=0;
- The labels of the fields as F:={1,2,3,⋯,i,⋯,n} with *n* number of fields;
- The known length of the growing season as D:={1,2,3,⋯,d,⋯,D} with *D* as the total number of days.

The term “area of interest (AOI)” is used in this paper to indicate a field of interest where fertilizer is to be applied.

## 2. Problem Description and Crop Model

One of the most critical decisions many farmers face on a daily basis is deciding which fields should receive N-fertilizer (also used as `fertilizer’ in this paper) and how much fertilizer to apply. The difficulty in making those decisions is due to a number of factors, including uncertainty regarding crop states, crop development, nitrogen supply from the soil, and future weather. Due to the unknown disturbances and uncertainties, farmers must rely on feedback and, thus, make decisions “online”. “Optimal” decision-making in the spread of N-fertilizers can then be defined in a variety of ways, depending on the user. Certain farmers may wish to maximize their economic profits by producing a large number of high-quality crops throughout the season. Other farmers may wish to produce a significant amount of crops with the least number of actions or by spreading the least amount of fertilizer in the field.

There are several methods for spreading fertilizer in a field, and in this paper, the utilization of spreader machines or a human operator is considered, with no limitation on the spreading capability that a machine (or manpower) has. The problem can be stated as “how to design an optimal automated decision support system for N-limited crops that considers the crop-growth dynamic model and predicts future actions”.

In this section, the crop model is discussed in nitrogen-limited situations, and it is assumed that there is no shortage of water. The LINTUL3 model describes crop nitrogen demand, uptake, and supply. The nitrogen nutrition index (NNI) measures the crop nitrogen deficit and lowers biomass production consequently.

### 2.1. Nitrogen Balance

Let $N_{i}^{d}$ represent the nitrogen content ($\mathrm{gN}\;m^{-2}$) in the soil of the field i∈F on day d∈D. According to the LINTUL3 [25,26], the modified dynamics of the soil–crop nitrogen balance can be written as

(1) $$\begin{array}{cc} N_{i}^{d+1} & =\left(N_{i}^{d}+u_{i}^{d}\right)\eta_{i}, \end{array}$$

where $u_{i}^{d}\in R_{\geq 0}$ is the nitrogen supply through mineralization (fertilization spreading) and $\eta_{i}\;\in \;\left[0,1\right]$ is the denitrification factor. It should be noted that N fertilization has some difficulties, such as soil degradation, which can make it challenging for plants to acquire the nutrients they demand [27,28]. Equation (1) can be further modified if the N-uptake by the plants is considered in the balanced equation. Further modifying the equation

(2) $$\begin{array}{cc} N_{i}^{d+1} & =\left(N_{i}^{d}+u_{i}^{d}\right)\eta_{i}-{\mathrm{NU}}_{i}^{d}, \end{array}$$

where ${\mathrm{NU}}_{i}^{d}$ is the rate of nitrogen uptake by the plant (rice crop) and is calculated by [29]

(3) $$\begin{array}{cc} {\mathrm{NU}}_{i}^{d} & =\min(\left(1\right),35.7{\left({\mathrm{LAI}}_{i}^{d}\right)}^{0.63}), \end{array}$$

where ${\mathrm{LAI}}_{i}^{d}$ represents leaf area index on day d∈D of the field i∈F.

The critical nitrogen concentration is the nitrogen concentration below which a crop experiences nitrogen stress. Nitrogen stress causes lower biomass production rates, which leads to lower yields. Nitrogen stress is thought to happen when the amount of nitrogen in the soil is much less than a critical value ${\mathrm{NCR}}_{i}^{d}=3.7{\left({\mathrm{LAI}}_{i}^{d}\right)}^{-0.35}$ for unrestricted growth [29]. The NNI, which goes from 0 (maximum N shortage) to 1 (no N shortage), is used to measure the lack of nitrogen:

(4) $$\begin{array}{cc} {\mathrm{NNI}}_{i}^{d} & =\frac{N_{i}^{d}}{{\mathrm{NCR}}_{i}^{d}}; \end{array}$$

consequently, the following constraint is introduced to ensure minimum stress

(5) $$\begin{array}{cc} 1-\delta & \leq {\mathrm{NNI}}_{i}^{d}, \end{array}$$

with suitable δ∈(0,1). It is worth noticing that ${\mathrm{NNI}}_{i}^{d}$ can even go above 1, which indicates the crop is not under the N stress and the soil is N rich.

### 2.2. Crop Growth Dynamics under N-Stress

The LINTUL3 crop growth dynamical model, for the case of flood irrigated rice, was considered for the analysis in this paper. The total biomass production (growth) above the ground $G_{i}^{d}$ in $g\;m^{-2}$ can be written in terms of intercepted irradiation as follows,

(6) $$\begin{array}{c} G_{i}^{d}=\frac{1}{2}\eta_{{\mathrm{LUE}}_{i}}{\mathrm{DTR}}_{i}^{d}\left(1-e^{-k{\mathrm{LAI}}_{i}^{d}}\right)e^{-\epsilon (1-{\mathrm{NNI}}_{i}^{d})}, \end{array}$$

where $\eta_{{\mathrm{LUE}}_{i}}$ represents the light use efficiency (LUE) and is in $g\;{\mathrm{MJ}}^{-1}$ (crop specific), ${\mathrm{DTR}}_{i}^{d}$ represents the total radiation in $\mathrm{MJ}\;m^{-2}\;d^{-1}$, *k* is the crop-specific attenuation coefficient in $m^{-2}\;m^{2}$, ${\mathrm{LAI}}_{i}^{d}$ is the leaf area index in $m^{2}\;m^{-2}$, ϵ is the LUE reduction factor under nitrogen stress. It is clear that if NNI=1 in (6), the maximum growth $G^{*}$ is attained. Additional information about the LINTUL3 model can be found in [26].

## 3. Optimization Problem Formulation

In order to begin the process of formulating the optimization problem, a cost term is defined in the following section.

### 3.1. Cost Function

In general, when managing crop fields, different farmers may be interested in pursuing different objectives, such as maximizing production growth and profits or allocating a minimum number of fertilizer distribution machines (or manpower), which minimizes labor or fertilizer costs. In this work, both aspects were considered. In particular, when coming to the cost of labor and fertilizer, related prices might vary over time, and, therefore, a good estimate of such quantities is an important requirement for the proposed work. The problem of estimating prices is not within the scope of this paper, where we assume such information is available. We instead point the interested reader to dedicated works in the literature [30,31]. The growth of the plants is dependent upon the desired quantity and quality of crops harvested at the end of each season.

Let α be the operation cost of the fertilizer spreading machine (or manpower) per day per field. This cost (which includes labor, fuel, and/or energy costs) needs to be paid to the assigned machine (or manpower). Let β be the resource cost that includes the cost per unit of the fertilizer. Then, the total cost of resources and operations per field i∈F per day d∈D will be

(7) $$\begin{array}{c} C_{i}^{d}=\left(\alpha_{i}^{d}+\beta_{i}^{d}\right)u_{i}^{d}. \end{array}$$

The potential biomass growth with no limitations on water and fertilizer is denoted by $G_{i}^{*d}$, and the actual growth under N-stress is denoted by $G_{i}^{d}$. To maximize the biomass growth, it is required to make the N-limited growth $G_{i}^{d}$ to track the potential growth $G_{i}^{*d}$ by minimizing

(8) $$\begin{array}{c} GR_{i}^{d}=\mathrm{abs}\left(G_{i}^{*d}-G_{i}^{d}\right). \end{array}$$

The maximum growth can be attained at $GR_{i}^{d}=0$.

### 3.2. Proposed Optimization Problem

Two optimization problems are discussed in this section. The variable $b_{i}^{d}\in B$ is the binary decision variable and if $b_{i}^{d}=1$, i.e., going high, this indicates that now it is the time to switch ON the fertilizer application (or else otherwise). The variable $u_{i}^{d}$ is the modulated control, which indicates the amount of fertilizer needed to be applied at the AOI. The relation between $b_{i}^{d}\in B$ and $u_{i}^{d}\in R_{\geq 0}$ is

(9) $$\begin{array}{c} b_{i}^{d}u_{\min}\leq u_{i}^{d}\leq b_{i}^{d}u_{\max}, \end{array}$$

where $u_{\min}$ and $u_{\max}$ are the minimum and maximum fertilizer quantities, respectively.

The following are the proposed optimization problems based on the growers’ decision.

(1) Maximum growth.
(2) Maximum growth considering the cost of labor and resources.

#### 3.2.1. Maximum Growth

In this paper, the term maximum growth refers to the maximum biomass growth, which can be achieved by tracking the potential growth of the biomass $G^{*}$, i.e., minimizing the distance between $G^{*}$ and *G*, without considering the cost of the use of fertilizer. The consequent optimization problem can be formulated using (8) as

(10) minb∈B,uid∈R≥0∑i∈F∑d∈DGRids.t.(2),(3),(4),(5),(9).

#### 3.2.2. Maximum Growth Considering the Cost of Labor and Resources

In order to maximize the biomass growth, growers should take into account the labor and fertilizer required to achieve it. The optimization problem can be formulated using (7) and (8) as

(11) minb∈B,uid∈R≥0∑i∈F∑d∈D(ω1Cid+ω2GRid)s.t.(2),(3),(4),(5),(9),

where in (11), $\omega_{1},\omega_{2}$ are positive weights that vary depending on the farmer’s priorities.

The next Algorithm 1 summarizes the procedure of the optimization framework.
**Algorithm 1** N-fertilizer application decision support system algorithm.1:Obtain the values of all the parameters2:Find the current state of $N_{i}^{0}$ in all *i* fields.3:**for**d = 1:D **do**4:    **for** i = 1:n **do**5:        Calculate ${\mathrm{LAI}}_{i}^{d}$, $\eta_{{\mathrm{LUE}}_{i}}$, ${\mathrm{DTR}}_{i}^{d}$, ${\mathrm{NCR}}_{i}^{d}$, ${\mathrm{NU}}_{i}^{d}$, ${\mathrm{NNI}}_{i}^{d}$, $G_{i}^{d}$6:        Minimize (10) or (11) subject to (2)–(5), (9)7:        Follow the instructions of $b_{i}^{d}$, $u_{i}^{d}$8:    **end for**9:**end for**

## 4. Numerical Simulations and Optimal Decision Support System

In this paper, two case studies are presented to solve the optimization problems. This section presents the numerical simulations based on the two optimization problems of (10) and (11). The simulation was done using MATLAB YALMIP [32] on an HP Z-Power, core i-7 machine with 32 GB of RAM.

### 4.1. Case 1: For Single Field

Consider solving the optimization problem for one field, i.e., n=1, with one machine (or manpower dedicated to that field). This optimization problem will be suitable for small farmers. The cost of labor is $€0.2\;{\mathrm{kg}}^{-1}$ and the cost of fertilizer is $€0.8\;{\mathrm{kg}}^{-1}$ [33].

- **Maximizing the growth**
  Consider the optimization problem of (10) and perform simulation, with the aim to increase the crop biomass growth without consideration of labor or resource costs.

**Figure 1:**
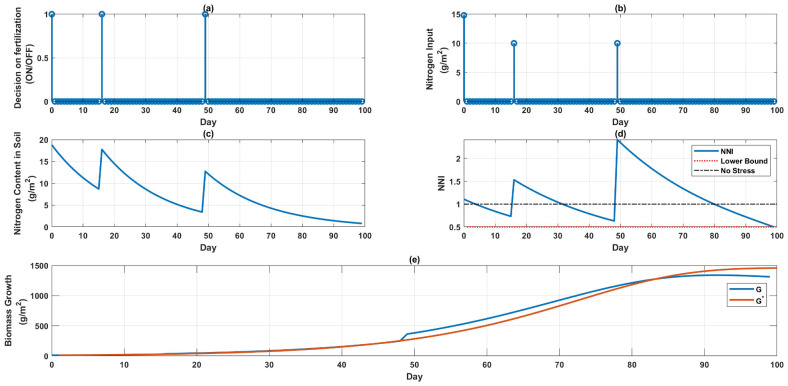
Maximizing the growth. (**a**) Indicates the days when fertilizer delivery is ON, (**b**) displays how much fertilizer is required on these days, (**c**) represents the nitrogen concentration of the soil, (**d**) the NNI level, and (**e**) the progressive development of biomass.

Figure 1 illustrates the simulation results for 100 days. It can be seen in Figure 1a that the fertilizer is supplied on days 1, 17, and 50; a total of three times in the whole lifetime of the crop growth, and more costs are paid on fertilization and labor. Figure 1b indicates the quantity of fertilizer needed. Figure 1c provides information about the quantity of fertilizer left in the soil, and Figure 1d indicates the NNI level. Figure 1e depicts the growth of the crop under fertilizer stress and the potential growth without stress. Figure 1 and Table 1 can be used straightforwardly to determine when and how much fertilizer to apply, and how much to pay on the resource and labor when targeting the maximum growth.

- **Maximum growth considering the cost of labor and resources**
  Consider the optimization problem of (11) and perform the simulation, with the aim to increase crop biomass growth with minimum labor or resource costs.

**Figure 2:**
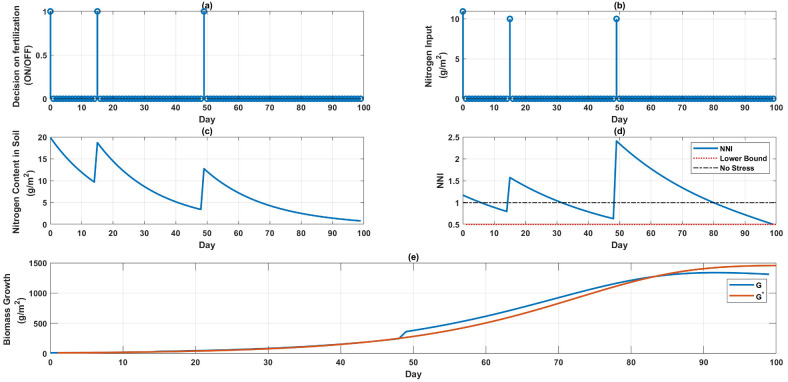
Maximum growth considering the cost of labor and fertilizer. (**a**) Indicates the days when fertilizer delivery is ON, (**b**) displays how much fertilizer is required on these days, (**c**) represents the nitrogen concentration of the soil, (**d**) the NNI level, and (**e**) the progressive development of the biomass.

Figure 2 shows the simulation of the total 100 days. It can be seen from Figure 2a that on the 1st, 16th, and 51st days, fertilization is required for targeting the maximum growth, taking into account the labor and resource costs. Figure 2b indicates the quantity of the fertilizer needed to be applied on AOI for these days; on the 1st, more fertilizer should be applied to obtain the maximum biomass growth. Figure 2 and Table 2 may be used to make the decision. The simulation provided relates to the farmer prioritizing labor and fertilizer costs by considering $\omega_{1}=100$, while also considering the highest biomass growth (with $\omega_{2}=1$).

Although there is no substantial difference between the parameters shown in Table 1 and Table 2 because the units are $m^{-2}$, this difference is big enough when scaled to a hectare.

### 4.2. Case 2: For Multiple Fields

In this section, we demonstrate that, if a farmer owns more than one field, it should first be determined how to choose the fertilizer, then it should be determined when and how much fertilizer should be applied to each field. Consider that a farmer has a sufficient number of fields in such a way that a fertilizer treatment on one field can leak through the slope and impact the dynamics of the nitrogen in other fields. This yields a more intriguing optimization problem.

The nitrogen dynamics will now change to

(12) $$\begin{array}{cc} N_{i}^{d+1} & =\left(N_{i}^{d}+u_{i}^{d}\right)\eta_{i}-{\mathrm{NU}}_{i}^{d}-\gamma_{i}N_{i}^{d}-\sum_{k\neq i}\zeta_{k}N_{k}^{d} \end{array}$$

where $\gamma_{i}$ and $\zeta_{k}$ are the exchange rates of nitrogen and k∈F. The optimization problems are now modified as

(13) minb∈B,uid∈R≥0∑i∈F∑d∈DGRids.t.(3)−(5),(9),(12),

to obtain the maximum biomass growth in multiple field cases, and

(14) minb∈B,uid∈R≥0∑i∈F∑d∈D(ω1Cid+ω2GRid)s.t.(3)−(5),(9),(12),

to obtain the maximum growth considering the cost of labor and resources in multiple field cases. The same algorithm of Algorithm 1 with modification in accordance to (13) and (14) can be utilized to find the optimum values. The computations are carried out by reducing the number of days in which it is necessary to apply the fertilizer; specifically, in this instance, it is considered that on day 1, the fertilizer needs to be applied (quantity not known), and the next fertilizer should be applied after the 10th day of the previous fertilization. This reduces the number of decision variables, which in turn reduces the time required to solve the optimization issue; hence, a mixed integer linear programming (MILP) problem is converted into a linear programming problem (LP). In the simulation, $\gamma_{i}$ and $\zeta_{i}$ have the following values: (i) Field 1: $\gamma_{1}=0.015$, $\zeta_{1}=0$, (ii) Field 2: $\gamma_{2}=0.015$, $\zeta_{1}=0.01$, and (iii) Field 3: $\gamma_{1}=0.01$, $\zeta_{1}=0.015$.

- **Maximizing the growth**
  This section is dedicated to solving the multi-field optimization problem to increase biomass growth. The fertilization treatment of Field 1 affects the nitrogen balance of Field 1 and Field 2; similarly, the fertilization treatment of Field 2 affects the nitrogen balance of Field 3.

**Figure 3:**
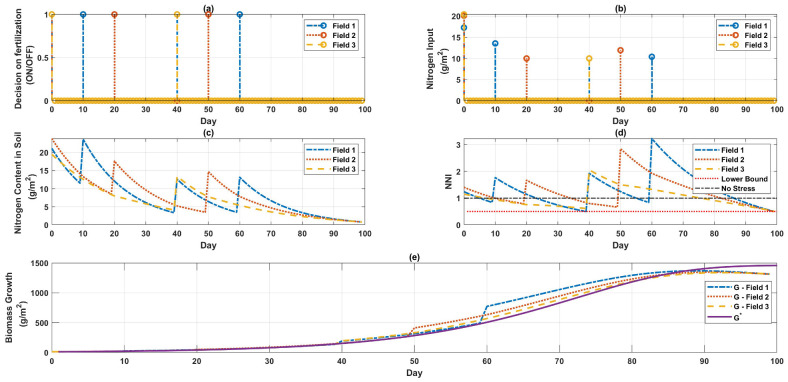
Maximizing the growth for multiple fields. (**a**) Indicates the days when fertilizer delivery is ON for the three fields, (**b**) displays how much fertilizer is required on these days in the three fields, (**c**) represents the nitrogen concentration of the soil within the three fields, (**d**) the NNI level, and (**e**) the progressive development of biomass in the three fields.

The timing of when to apply the fertilizer and the quantity of fertilizer that should be applied are shown in Figure 3 and same is summarized in Table 3. Figure 3a provides information regarding the days on which fertilization needs to be turned on in AOI, and Figure 3b specifies the quantity of fertilizer required to be added to AOI on these days.

- **Maximum growth considering the cost of labor and resources**
  This section is dedicated to solving the multi-field optimization problem to maximize biomass growth considering the cost of labor and resources.

**Figure 4:**
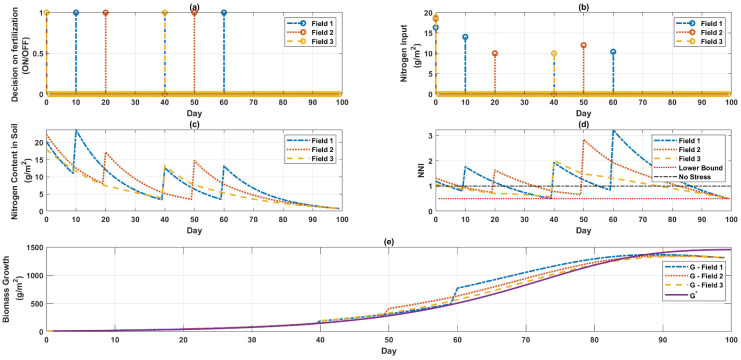
Maximum growth considering the cost of labor and resources for multiple fields. (**a**) Indicates the days when fertilizer delivery is ON for the three fields, (**b**) displays how much fertilizer is required on these days in the three fields, (**c**) represents the nitrogen concentration of the soil within the three fields, (**d**) the NNI level, and (**e**) the progressive development of the biomass in the three fields.

Figure 4 shows when and how much fertilizer should be used in which field. Figure 4a provides information regarding the days on which fertilization needs to be turned on in AOI and Figure 4b specifies the quantity of fertilizer required to be added to AOI on these days. Figure 4 and Table 4 can be used for optimal decision-making and saving some costs while maximizing biomass growth.

## 5. Results and Discussion

The harvest index (HI) is defined as

(15) $$\begin{array}{c} \mathrm{HI}=\frac{\mathrm{Grain}\;\mathrm{Yield}}{\mathrm{Biomass}\;\mathrm{Growth}}, \end{array}$$

and for rice, this quantity is 0.46 to 0.50 [34]. This relation is used to find the grain yield in this section. Table 5 provides a comparison between the results obtained from the proposed work with the existing literature.

The optimization problem of just one field was investigated in order to facilitate a fair comparison of the outcomes obtained from the simulation. It is possible to conclude from the findings reported in Table 5 that the optimization problem addressed in this study achieves its desired goals and, in some cases, performs well enough when compared to the findings presented in the relevant literature review. However, there might be some limitations that may occur due to; (i) the uncertainty in the measurements from the sensors, i.e., when measuring N-fertilizer availability, the result depends on the sensor system, measurement process, or other environmental conditions. Even though the quantity is measured repeatedly in the same way and under the same conditions, each time a different value is obtained, (ii) from the commercial point of view, making the system fully automated puts an additional load on the farmers. Such limitations will need to be taken properly into account in the future.

The treatments for fertilizing $300\;\mathrm{kgN}\;{\mathrm{ha}}^{-1}$ of the proposed work have shown good results when compared to other relevant works in the literature. Specifically, when compared to [35], the same amount of N-fertilizer applied results in a 28% increase in the amount of biomass that could be produced. When compared to [36], it is possible to obtain a 52% increase in biomass output by applying 33.33% less of the fertilizer that was used in [36]. When compared to [37], a 37% increase in biomass output may be accomplished with a 33.33% lower amount of fertilizer application, w.r.t. [37].

It has also been demonstrated that high nitrogen treatments made during the early vegetative stage were critical indicators of increasing growth and production [17]; this assertion was confirmed once again by considering the outcomes of the optimization depicted in Figure 1, Figure 2, Figure 3 and Figure 4. After day 61, nitrogen fertilization was not required in the case of a single field or in the case of multiple fields.

## 6. Conclusions and Future Work

An optimization problem is addressed in this article. The results of the analysis proved helpful in determining when and how much nitrogen fertilizer should be given to a particular AOI. Specifically, considering the LINTUL3 plant dynamic model, the optimization strategy addressed in this manuscript proved useful in the nitrogen management of the flooded irrigated rice crop. Furthermore, the results of the simulation were compared with those that were found in the existing literature. It was discovered that, in some cases, when the suggested work was utilized for optimal nitrogen management for a crop (in the paper, it was rice), it experienced a 28% to 53% improvement in biomass growth. In future work, the primary objective will be to experimentally validate the findings of the optimal nitrogen management strategy, either for a single field or for multiple fields simultaneously. The objective will be to alter the soil characteristics by adding the foreign element, taking into account the impact on crop growth.

## Figures and Tables

**Table 1:** Case 1 result: Maximizing the growth.

Initial N-Content ($g\;m^{-2}$)	Decision Based on *b* (ON Day (D))	N-Supply through Fertilization ($g\;m^{-2}$)	Total Cost on N ($\;m^{-2}$) Fertilizer	Cost on Labor ($\;m^{-2}$)	Biomass Growth ($g\;m^{-2}$)
5	D: 1, 17, 50	D1: 14.801, D17: 10, D50: 10	0.0278	0.0069	1320

**Table 2:** Case 1 result: Maximum growth considering the cost of labor and fertilizer.

Initial N-Content ($g\;m^{-2}$)	Decision Based on *b* (ON Day (D))	N-Supply through Fertilization ($g\;m^{-2}$)	Total Cost on N ($\;€\;m^{-2}$) Fertilizer	Cost on Labor ($\;€\;m^{-2}$)	Biomass Growth ($g\;m^{-2}$)
5	D: 1, 16, 51	D1: 10, D16: 10, D51: 10	0.0240	0.0060	1312

**Table 3:** Case 2 result: Maximizing the growth.

Field No.	Initial N-Content ($g\;m^{-2}$)	Decision Based on *b* (ON Day (D))	N-Supply through Fertilization ($g\;m^{-2}$)	Total Cost on N ($\;€\;m^{-2}$) Fertilizer	Cost on Labor ($\;€\;m^{-2}$)	Biomass Growth ($g\;m^{-2}$)
1	5	D: 1, 11, 41, 61	D1: 17.298, D11: 13.556, D41: 10, D61: 10.398	0.0410	0.0102	1312
2	5	D: 1, 21, 51	D1: 20.121, D21: 10, D51: 11.949	0.0336	0.0084	1312
3	0	D: 1, 41	D1: 20.431, D41: 10	0.0243	0.0060	1316.4

**Table 4:** Case 2 result: Maximum growth considering the cost of labor and resources for multiple fields.

Field No.	Initial N-Content ($g\;m^{-2}$)	Decision Based on *b* (ON Day (D))	N-Supply through Fertilization ($g\;m^{-2}$)	Total Cost on N ($\;€\;m^{-2}$) Fertilizer	Cost on Labor ($\;€\;m^{-2}$)	Biomass Growth ($g\;m^{-2}$)
1	5	D: 1, 11, 41, 61	D1: 16.353, D11: 14.038, D41: 10, D61: 10.398	0.0406	0.0101	1312
2	5	D: 1, 21, 51	D1: 18.516, D21: 10, D51: 12.008	0.0324	0.0081	1312
3	0	D: 1, 41	D1: 18.839, D41: 10	0.0230	0.0057	1315.3

**Table 5:** Results comparison.

*N*-Fertilizer Input ($\mathrm{kg}\;{\mathrm{ha}}^{-1}$)	Application	Final Biomass ($t\;{\mathrm{ha}}^{-1}$)	Final Yield ($t\;{\mathrm{ha}}^{-1}$)	Reference
300 (30 $g\;m^{2}$)	optimal nitrogen management	9.5–16.3 (950–1630 $g\;m^{2}$)	5.4–9.0 (540–900 $g\;m^{2}$)	[35]
400 (40 $g\;m^{2}$)	nitrogen management	6.3–16.1 (630–1610 $g\;m^{2}$)	3.4–6.7 (340–670 $g\;m^{2}$)	[36]
400 (40 $g\;m^{2}$)	nitrogen management	8.3–18.2 (830–1820 $g\;m^{2}$)	4.8–10.1 (480–1010 $g\;m^{2}$)	[37]
225 (22.5 $g\;m^{2}$)	nitrogen management	10–18.2 (1000–1820 $g\;m^{2}$)	5.6–9.9 (560–990 $g\;m^{2}$)	[38]
225 (+150 K ${}^{1}$ and 90 P ${}^{2}$) (22.5 $g\;m^{2}$)	nitrogen management	19.45 (1945 $g\;m^{2}$)	10.43 (1043 $g\;m^{2}$)	[39]
170 (17 $g\;m^{2}$)	nitrogen management	9.52 (952 $g\;m^{2}$)	3.47 (347 $g\;m^{2}$)	[40]
300–348 ${}^{3}$ (30–34.8 $g\;m^{2}$)	optimal nitrogen management	13.12–13.20 (1312–1320 $g\;m^{2}$)	6.6–6.81 (660–681 $g\;m^{2}$)	Proposed Work

K ^1^: Potassium, P ^2^: Phosphorous; () ^3^: HI = 0.5, optimization problem of one field has been considered to compare the results.

## Data Availability

Not applicable.

## Abbreviations

The following abbreviations are used in this manuscript:

LUE	light use efficiency ($g\;{\mathrm{MJ}}^{-1}$)
NNI	Nitrogen Nutrition Index
NU	nitrogen uptake ($\mathrm{gN}\;m^{-2}$)
LAI	leaf area index ($m^{2}m^{-2}$)
DTR	daily total radiation ($\mathrm{MJ}\;m^{-2}$)
MILP	mixed integer linear programming
LP	linear programming problem
AOI	area of interest
